# Inflammatory Cytokines Impair Glucagon Expression and Secretion in Pancreatic α‐Cells

**DOI:** 10.1111/dom.70921

**Published:** 2026-05-31

**Authors:** Caroline Bickelmann, Cedric Wilden, Leticia Prates‐Roma, Matthias W. Laschke, Emmanuel Ampofo, Selina Wrublewsky

**Affiliations:** ^1^ Institute for Clinical and Experimental Surgery, Saarland University, PharmaScienceHub (PSH) Homburg Germany; ^2^ Center for Gender Specific Biology and Medicine (CGBM) Saarland University Homburg Germany; ^3^ Biophysics Department, Center for Human and Molecular Biology (ZHMB) Saarland University Homburg Germany

**Keywords:** cytokines, glucagon, inflammation, sex, type‐2 diabetes mellitus

## Abstract

**Aims:**

Insulin resistance and obesity‐associated inflammation are key drivers in the pathogenesis of Type 2 diabetes mellitus (T2DM). Whilst inflammatory cytokines are well known to impair β‐cell function, their impact on pancreatic α‐cells and glucagon (GCG) regulation remains poorly understood. In this study, we investigated the effects of the pro‐inflammatory cytokines interleukin (IL)‐1β, tumour necrosis factor (TNF)‐α and interferon (IFN)‐γ on GCG expression and secretion.

**Materials and Methods:**

The viability and endocrine function of α‐cell line αTC1 and isolated islets were investigated by WST‐1 assay, LDH assay, qRT‐PCR, Western blot analysis and ELISA. The transcriptional activity of the GCG promoter was analysed by reporter gene assays. The cellular composition of isolated islets was assessed by immunohistochemistry.

**Results:**

We found that exposure of the α‐cell line αTC1 to a mix of these cytokines activates cellular stress responses characterised by induction of the nuclear factor kappa‐light‐chain‐enhancer of activated B‐cells (NF‐κB) pathway and the nuclear factor erythroid 2‐related factor 2 (Nrf2) pathway. Moreover, cytokine treatment markedly reduced GCG gene expression and secretion through repression of GCG promoter activity. Mechanistically, this was associated with a disrupted transcriptional network. These findings were confirmed in isolated mouse islets, where cytokine exposure significantly reduced GCG expression and secretion in islets of both male and female donors.

**Conclusions:**

Taken together, these findings indicate that inflammatory cytokines are potent modulators of α‐cell function as well as GCG secretion and provide novel insights into inflammation‐driven dysregulation of the endocrine function of pancreatic islets.

## 1 Introduction

Insulin resistance is defined as a reduced responsiveness of target cells to insulin‐mediated glucose uptake and is considered the predominant driving factor throughout the transition from prediabetes to manifest Type 2 diabetes mellitus (T2DM). In response to insulin resistance, pancreatic islets expand their functional mass and increase their insulin secretion [1].

Despite extensive research, the mechanistic basis of insulin resistance remains incompletely understood. The identification of elevated circulating inflammatory markers, such as C‐reactive protein, chemokines and cytokines, as well as increased tumour necrosis factor (TNF)‐α levels in adipose tissue of patients with T2DM has established a link between obesity, insulin resistance and islet inflammation [2]. In fact, this link has reshaped the current understanding of T2DM pathophysiology and highlights obesity‐induced inflammation as a critical contributor to disease progression [3].

Several studies already reported that inflammatory cytokines inhibit β‐cell proliferation by promoting the release of chemokines, trigger β‐cell dedifferentiation and promote the formation of reactive oxygen species (ROS) [3, 4, 5, 6, 7]. Mechanistic analyses revealed that this inflammatory response is mediated by various pathways, including the nuclear factor kappa‐light‐chain‐enhancer of activated B‐cells (NF‐κB) pathway and the nuclear factor erythroid 2‐related factor 2 (Nrf2) pathway, a key regulator of cellular anti‐oxidant responses [8]. The cross‐talk of both pathways is responsible for maintaining redox balance in β‐cells and any disruption of this balance can lead to impaired insulin secretion mediated by reduced expression of pancreatic and duodenal homeobox 1 (PDX1) and v‐maf avian musculoaponeurotic fibrosarcoma oncogene homologue A (MafA) [7, 8, 9]. Accordingly, treatment of patients with anti‐inflammatory drugs may improve hyperglycemia in T2DM [10].

In contrast, little is known about the effect of inflammatory cytokines on other endocrine pancreatic hormones, such as glucagon (GCG). A few studies reported that interleukin (IL)‐1 signalling exerts inhibitory effects on GCG secretion [11, 12, 13]. However, the underlying molecular mechanisms are still unknown. Therefore, the aim of the present study was to analyse the effects of a mix of the inflammatory cytokines IL‐1β, TNF‐α and interferon (IFN)‐γ on GCG gene expression and secretion.

## 2 Material and Methods

### 2.1 Materials

Collagenase NB 8 was purchased from Nordmark (Uetersen, Germany). Neutral red solution, Tween20 and Hoechst 33342 were purchased from Sigma‐Aldrich (Taufkirchen, Germany). IL‐1β, TNF‐α and IFN‐γ were purchased from Thermo Fisher Scientific (Dreieich, Germany). Bovine serum albumin (BSA) and foetal calf serum (FCS) were purchased from Santa Cruz Biotechnology (Heidelberg, Germany). Cell lysis reagent QIAzol and QuantiNova Reverse Transcription Kit were purchased from Qiagen (Hilden, Germany). Annexin‐V‐FLUOS staining kit, HepatoQuick, water‐soluble tetrazolium (WST)‐1 and lactate dehydrogenase (LDH) assays were purchased from Roche (Basel, Switzerland). The qScriber cDNA Synthesis Kit and ORA SEE qPCR Green ROX L Mix were purchased from HighQu (Kraichtal, Germany). Protein assay dye reagent and luminol‐enhanced chemiluminescence (ECL) Western blotting substrate were purchased from Bio‐Rad Laboratories (Feldkirchen, Germany). Lipofectamine 3000, Dulbecco's Modified Eagle Medium (DMEM; 1 g glucose/L [5.5 mmol/L]) and Roswell Park Memorial Institute (RPMI) 1640 medium (2 g glucose/L [11 mmol/L]) were purchased from Fisher Scientific (Schwerte, Germany). Ketamine (Ketabel) was purchased from Bela‐Pharm GmbH & Co. KG (Vechta, Germany). Xylazine (Rompun) was purchased from Bayer (Leverkusen, Germany). GCG enzyme‐linked immunosorbent assay (ELISA) kit (DGCG0) was purchased from R&D Systems (Minneapolis, USA). Bay 11‐7082 was purchased from MedChemExpress (Sollentuna, Sweden). Propidium iodide was purchased from BD Biosciences (San Jose, USA). Accutase was purchased from BioLegend (Koblenz, Germany).

### 2.2 Antibodies

Anti‐insulin (66198), anti‐β‐actin (HRP‐66009), anti‐GCG (15954), anti‐p65 (80979), anti‐Nrf2 (16396) and anti‐GAPDH (HRP‐60004) antibodies were purchased from Proteintech (Manchester, UK). Anti‐somatostatin (SST) (4770) and anti‐superoxide dismutase 2 (SOD2) (137254) antibodies were purchased from Santa Cruz (Heidelberg, Germany). Anti‐phospho (p)‐p65 antibody (3033) was purchased from cell signalling (Danver, USA). Anti‐cyclooxygenase (COX)2 (ab15191) and anti‐inducible nitric oxide synthase (iNOS) (ab15323) antibodies were purchased from abcam (Cambridge, England). Anti‐heme oxygenase (HO)‐1 (ADI‐SPA‐895‐F) antibody was purchased from Enzo Life Science (Farmingdale, USA). Peroxidase‐labelled anti‐rabbit (NIF 824) and peroxidase‐labelled anti‐mouse (NIF 825) antibodies were purchased from GE Healthcare (Freiburg, Germany).

### 2.3 Cell Culture

The murine pancreatic α‐cell line αTC1 clone 6 (ATCC: CRL‐2934) was cultivated in DMEM (1 g/L glucose) supplemented with 10% (v/v) FCS in a humidified atmosphere with 5% CO_2_ at 37°C. The αTC1 cells were exposed to a cytokine mix (2 ng/mL IL‐1β, 10 ng/mL TNF‐α and 50 ng/mL IFN‐γ) or the equivalent volume of phosphate‐buffered saline (PBS; vehicle) as control for 24 h. The combination of these cytokines in different concentrations is widely used for mimicking inflammatory cytokine stress [5, 14]. For the inhibition of the NF‐κB pathway, α‐cells were pretreated with the NF‐κB inhibitor bay 11‐7082 (5 μM for 1 h) prior to cytokine exposure for 24 h. All experiments were carried out with confluent cells between the third and seventh passage. The cells were passaged at a split ratio of 1:3 after reaching confluence.

### 2.4 WST‐1 Assay

αTC1 cells were seeded in a 96‐well plate and exposed to cytokine mix or vehicle for 24 h. Thereafter, a WST‐1 assay kit was used to measure the metabolic activity of the treated cells according to the manufacturer's instructions.

### 2.5 LDH Assay

αTC1 cells were seeded in a 96‐well plate and exposed to cytokine mix or vehicle for 24 h. Thereafter, an LDH assay kit was used to analyse the cytotoxic effects of the cytokines IL‐1β, TNF‐α and IFN‐γ on αTC1 cells according to the manufacturer's instructions.

### 2.6 Flow Cytometry

For propidium iodide/annexin V stainings, isolated islets were dispersed into single cells by accutase. Subsequently, the cells were washed in PBS, resuspended in the incubation buffer and stained for 15 min with propidium iodide and annexin V according to the manufacturer's protocol. The stained cells were analysed by flow cytometry using a FACSLyric flow cytometry system (BD Biosciences) and the fractions of vital, apoptotic, necrotic as well as necroptotic cells were given in % of all measured cells.

### 2.7 Western Blot Analysis

For the generation of whole cell extracts, αTC1 cells or islets (exposed to cytokine mix or vehicle for 24 h) were lysed for 30 min at 4°C with lysis buffer (10 mmol/L Tris–HCl, pH 7.5, 10 mmol/L NaCl, 0.1 mmol/L EDTA, 0.5% (v/v) Triton X‐100, 0.02% (w/v) NaN_3_) supplemented with 0.5 mmol/L phenylmethylsulfonyl fluoride (PMSF). The cell extracts were generated and analysed as described previously in detail [15]. After electrophoresis and blotting onto polyvinylidene difluoride (PVDF) membranes by a semi‐dry blot procedure (BioRad, Munich, Germany), the membranes were blocked using Tris‐buffered saline (TBS; 20 mM Tris–HCl, pH 7.5, 150 mM NaCl) supplemented with 0.1% (v/v) Tween20 (TBS‐T) and 5% BSA (w/v) for 1 h at room temperature (RT). Primary antibodies were diluted 1:1000 with TBS‐T and 1% BSA (w/v) and incubated for 1 h at RT. Subsequently, membranes were washed twice with TBS‐T and then incubated with specific secondary antibodies (dilution of 1:10 000 in TBS‐T and 1% BSA (w/v) for 1 h at RT). After washing, the expression of the proteins was visualised by enhanced chemiluminescence using the ECL Western blotting substrate.

### 2.8 Quantitative Real Time‐Polymerase Chain Reaction (qRT‐PCR)

Total RNA from αTC1 cells or islets (exposed to cytokine mix or vehicle for 24 h) was isolated using QIAzol lysis reagent. The corresponding cDNA was synthesised from 1 μg of total RNA by QuantiNova Reverse Transcription Kit according to the manufacturer's instructions. ORA qPCR Green ROX L Mix was used for qRT‐PCR. The data analysis was performed by the MiniOpticon Real‐Time PCR System. Murine β‐actin served as internal control for mRNA detection. Forward and reverse primers were used in a concentration of 700 nM solved in RNase/DNase‐free H_2_O. Primer sequences for qPCR were coded as follows: mouse GCG forward 5′‐TGGACTCCCGCCGTGCTCAAG‐3′, reverse 5′‐CCTTTGCTGCCTGGCCCTCC‐3′; mouse PDX1 forward 5′‐GAAATCCACCAAAGCTCACG‐3′, reverse 5′‐GAATTCCTTCTCCAGCTCCAG‐3′; mouse paired box protein pax 6 (PAX6) forward 5′‐CCCATGCAGATGCAAAAGTC‐3′, reverse 5′‐CAGTCTCGTAATACCTGCCC‐3′; mouse v‐maf avian musculoaponeurotic fibrosarcoma oncogene homologue B (MafB) forward 5′‐TACTGGATGGCGAGCAACTACC‐3′, reverse 5′‐ACTACGGAAGCCGTCGAAGCTC‐3′ and mouse β‐actin forward 5′‐CCTAGGCACCAGGGTGTGAT‐3′, reverse 5′‐TCTCCATGTCGTCCCAGTTG‐3′.

### 2.9 Reporter Luciferase Assay

The sequence of the mouse GCG promoter was amplified and the resulting construct was cloned into the XhoI restriction site of the luciferase reporter vector pGL4.10 (Promega, Mannheim, Germany). The identity of pGL4.10‐GCG was verified by sequencing. The transcriptional activity of the GCG promoter was assessed by reporter gene assays according to the manufacturer's instructions (Promega, Mannheim, Germany). Briefly, αTC1 cells were seeded in a 24‐well plate. Subsequently, the cells were transfected with the pGL4‐GCG reporter vector by using Lipofectamine 3000 for 24 h. In addition, pGL4‐GCG‐transfected cells were exposed to cytokine mix or vehicle for 24 h. Then, the cells were lysed and the luciferase activity was detected by a luminescence plate reader.

### 2.10 ELISA

The amount of secreted GCG was determined by a GCG ELISA kit. For this purpose, αTC1 cells or islets were cultivated in krebs ringer buffer (KRB; 115 mM NaCl, 4.7 mM KCl, 1.28 mM CaCl_2_, 1.2 mM MgSO_4_, 0.1% BSA, 25 mM glucose) for 1 h at 37°C and 5% CO_2_. Subsequently, the buffer was removed and αTC1 cells or islets were cultivated in KRB containing 0.5 mM or 11 mM glucose for 2 h. The supernatants were collected and the amount of secreted GCG was determined by using a GCG ELISA kit according to the manufacturer's protocol.

### 2.11 Animals

Male and female C57BL/6J mice with a body weight of 25–30 g served as donors for islet isolation. The animals were maintained on a standard 12/12 h day/night cycle. Water and standard pellet chow (ssniff Spezialdiäten GmbH, Soest, Germany) were provided ad libitum. All experiments were performed according to the German legislation on protection of animals and the National Institutes of Health (NIH) Guide for the Care and Use of Laboratory Animals (Institute of Laboratory Animal Resources, National Research Council, Washington DC, USA). The experiments were approved by the local authorities (State Office for Consumer Protection, Saarbrücken, Germany).

### 2.12 Isolation of Pancreatic Islets

Mice were anaesthetised by intraperitoneal (i.p.) injection of ketamine (100 mg/kg body weight) and xylazine (12 mg/kg body weight). Following cervical dislocation and midline laparotomy, the pancreatic duct was injected with 1 mg/mL collagenase NB 8 containing 25 μL/mL neutral red solution. The pancreas was excised and further digested by collagenase (collagenase NB 4G; 1 mg/mL). After washing with PBS containing 10% FCS to inactivate the collagenase, the islets were purified by hand picking. The isolated islets were cultivated in RPMI (supplemented with 10% (v/v) FCS, 100 U/mL penicillin and 0.1 mg/mL streptomycin) for 24 h at 37°C and 5% CO_2_ for further experiments.

### 2.13 Immunohistochemistry

Islets (exposed to cytokine mix or vehicle for 24 h) were incubated for 45 min at 37°C in 100 μL HepatoQuick, 50 μL human citrate plasma and 10 μL 10% CaCl_2_ solution. The resulting clot was also fixed for 24 h in 4% PFA. The PFA‐fixed specimens were embedded in paraffin and 3‐μm‐thick sections were cut. To study the cellular composition, the sections were stained with antibodies against insulin (β‐cells), GCG (α‐cells) as well as SST (δ‐cells) and visualised by their corresponding secondary antibodies. Cell nuclei were stained with Hoechst 33342. The sections were analysed by means of a BX60F microscope (Olympus, Evident Europe, Hamburg, Germany). The quantification of positively stained cells was done by the FIJI software (NIH) and is given in % of all islet cells.

### 2.14 Statistical Analysis

For testing the data for normal distribution and equal variance, we performed the Shapiro–Wilk test on the raw and the normalised values. Differences between two normally distributed groups were assessed by the unpaired Student's *t*‐test. To test differences between multiple normally distributed groups, one‐way ANOVA was applied following the Tukey post hoc test. If we compared two non‐normally distributed groups, the Mann–Whitney *U* test was used. If we compared three non‐normally distributed groups, we performed ANOVA on ranks (non‐parametric test: Kruskal–Wallis) following Dunn's post hoc test. The statistical analysis was performed by means of Prism software 10.6.1 (GraphPad, San Diego, USA). The results were expressed as mean ± standard error of the mean (SEM). *p*‐values < 0.05 indicated statistical significance.

## 3 Results

### 3.1 Inflammatory Cytokines Activate Stress Programmes in α‐Cells Without Affecting Cell Viability

**FIGURE 1:**
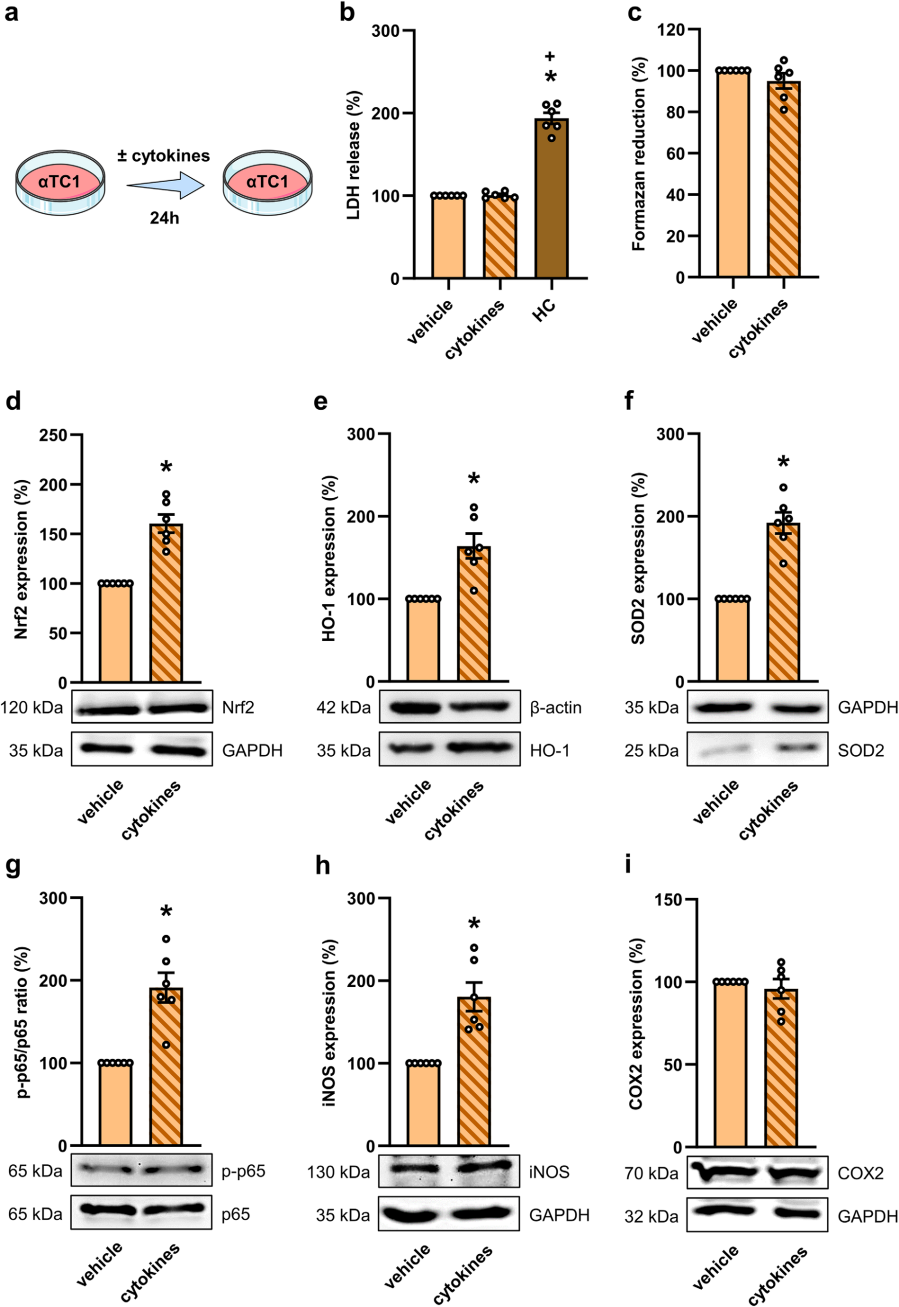
Inflammatory cytokines activate stress programmes in α‐cells without affecting cell viability. (a) Schematic illustration of the experimental setting: αTC1 cells were exposed to a cytokine mix (2 ng/mL IL‐1β, 10 ng/mL TNF‐α and 50 ng/mL IFN‐γ) or the equivalent volume of PBS (vehicle) as control for 24 h. (b) αTC1 cells were exposed to a cytokine mix or vehicle for 24 h and the cytotoxicity was assessed by a LDH assay. Cells were permeabilized with Triton‐X100 (0.1%) and used as high toxicity control (HC) for the LDH assay. Data are expressed in % to vehicle‐treated cells (*n* = 6 each). Mean ± SEM. *p < 0.05 versus vehicle. ^+^ *p* < 0.05 versus cytokines. (c) αTC1 cells were exposed to a cytokine mix or vehicle for 24 h and the mitochondrial activity was analysed by a WST‐1 assay. Data are expressed in % to vehicle‐treated cells (*n* = 6 each). Mean ± SEM. (d–i) Upper panel: Nrf2 (D), HO‐1 (e), SOD2 (f), p‐p65/p65 ratio (g), iNOS (h) and COX2 (i) protein expression in αTC1 cells exposed to a cytokine mix or vehicle for 24 h. Data are expressed in % of vehicle‐treated cells (*n* = 6 each). Mean ± SEM. *p < 0.05 versus vehicle. Lower panel: Representative Western blots of Nrf2, HO‐1, SOD2, p‐p65/p65, iNOS, COX2, GAPDH and β‐Actin expression in whole cell extracts.

Inflammatory islet studies have previously shown that exposure to IL‐1β, TNF‐α and IFN‐γ induces ROS‐mediated cell death [5]. To ensure that the used cytokine mix does not lead to activation of cell death signalling pathways, we exposed the α‐cell line αTC1 to these molecules for 24 h and assessed cellular viability as well as the expression of ROS‐associated stress markers (Figure 1a–i). Neither LDH release nor WST‐1 measurements revealed the induction of cell death following cytokine treatment (Figure 1b,c). In contrast, cytokine exposure led to significantly elevated levels of the stress‐responsive transcription factor Nrf2 (Figure 1d), which regulates genes encoding anti‐oxidant enzymes via anti‐oxidant response elements (ARE). Accordingly, expression of the anti‐oxidant enzymes HO‐1 and SOD2 was significantly increased in cytokine‐treated cells when compared to vehicle‐treated controls (Figure 1e,f). In addition, we analysed the activation of the NF‐κB pathway, which induces the transcription of pro‐inflammatory mediators, such as iNOS and COX2. As expected, cytokine‐treated α‐cells exhibited increased NF‐κB activity as shown by an elevated iNOS expression (Figure 1g,h). However, expression of COX2 was not affected when compared to vehicle‐treated control (Figure 1i).

### 3.2 Inflammatory Cytokines Disrupt the GCG Transcriptional Network

**FIGURE 2:**
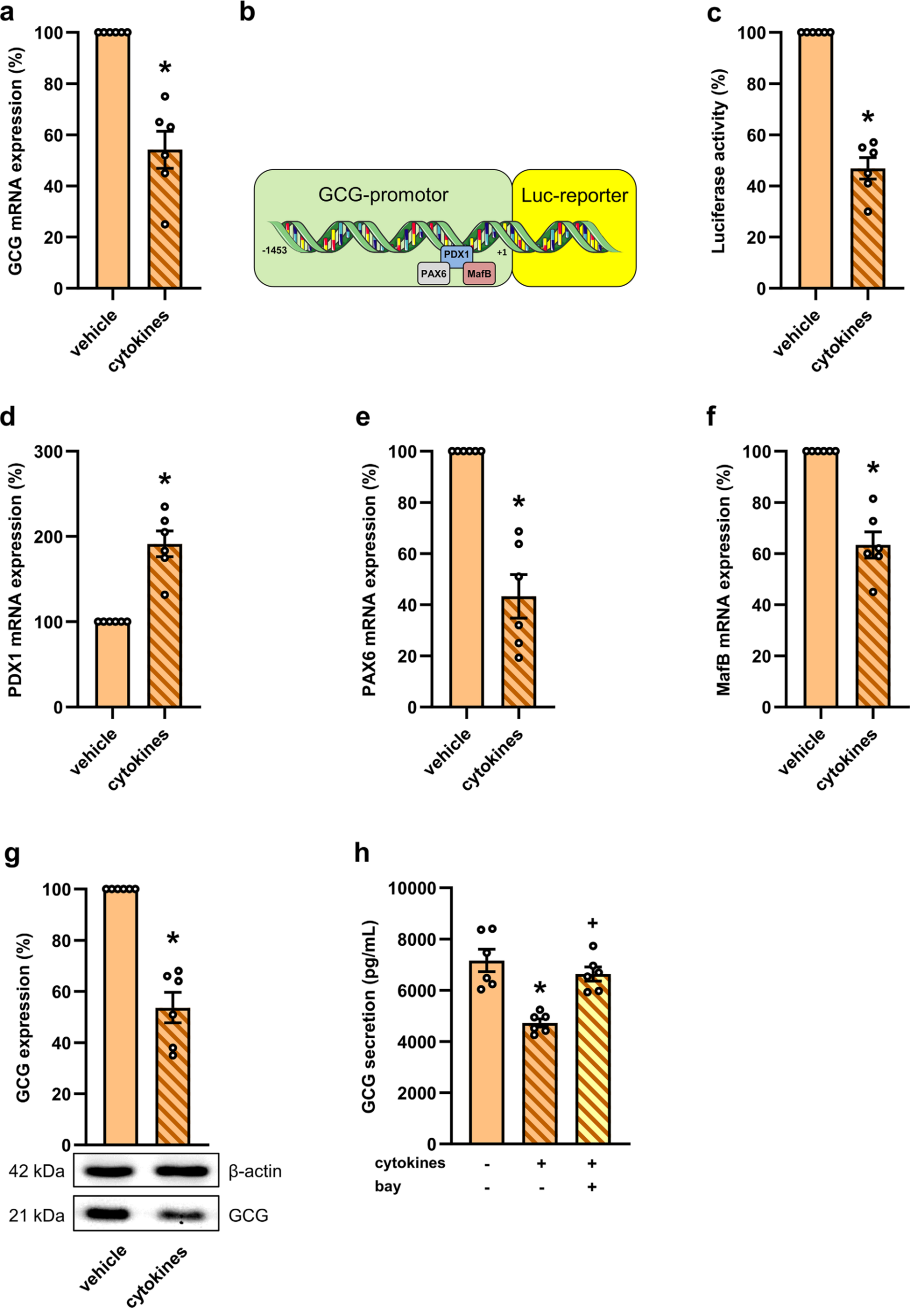
Inflammatory cytokines disrupt the GCG transcriptional network. (a) GCG mRNA expression in αTC1 cells exposed to a cytokine mix or vehicle for 24 h. Data are expressed in % to vehicle‐treated cells (*n* = 6 each). Mean ± SEM. *p < 0.05 versus vehicle. (b) Schematic illustration of the GCG‐promoter construct with the overlapping PDX1, PAX6 and MafB binding sites. (c) αTC1 cells were transfected with pGL4‐GCG and exposed to a cytokine mix or vehicle for 24 h. Then, the cells were lysed and the promoter activity was detected by a luciferase assay (*n* = 6 each). Mean ± SEM. *p < 0.05 versus vehicle. (d–f) PDX1 (d), PAX6 (e) and MafB (f) mRNA expression in αTC1 cells exposed to a cytokine mix or vehicle for 24 h. Data are expressed in % to vehicle‐treated cells (*n* = 6 each). Mean ± SEM. *p < 0.05 versus vehicle. (g) Upper panel: GCG protein expression in αTC1 cells exposed to a cytokine mix or vehicle for 24 h. Data are expressed in % of vehicle‐treated cells (*n* = 6 each). Mean ± SEM. *p < 0.05 versus vehicle. Lower panel: Representative Western blots of GCG and β‐Actin expression in whole cell extracts. (h) GCG secretion (pg/mL) from cells exposed to vehicle, a cytokine mix or a combination of bay 11‐7082 and cytokine mix (*n* = 6 each). Mean ± SEM. *p < 0.05 versus vehicle. ^+^ *p* < 0.05 versus cytokines.

We next examined whether the cytokine mix affects GCG expression and secretion. Of interest, cytokine treatment markedly reduced GCG mRNA levels in α‐cells (Figure 2a), indicating repression at the transcriptional level. In fact, promoter‐reporter assays confirmed that IL‐1β, TNF‐α and IFN‐γ directly impact GCG promoter activity (Figure 2b,c).

To identify the underlying transcriptional mechanisms, we analysed the expression of the key transcription factors PDX1, PAX6 and MafB that regulate GCG gene expression. Our results showed that the cytokines significantly upregulated PDX1 mRNA expression (Figure 2d), whereas the expression of PAX6 and MafB was significantly decreased (Figure 2e,f). This transcriptional shift resulted in reduced GCG protein expression and subsequently decreased GCG secretion when compared to control (Figure 2g,h). To confirm that these observations are due to activation of the NF‐κB pathway, α‐cells were treated with the NF‐κB inhibitor bay prior to cytokine exposure. We found that bay suppresses cytokine‐induced iNOS expression (Figure S1a). Furthermore, bay pretreatment abolished the cytokine‐induced changes in PDX1, PAX6 and MafB mRNA expression and thus increases GCG expression and secretion (Figures S1b–f and 2h).

To assess the effects of the individual cytokines, α‐cells were exposed to IL‐1β, TNF‐α or IFN‐γ, showing that all three cytokines independently exert inhibitory effects on GCG secretion (Figure S1g). Moreover, we demonstrated that the cytokine mix also suppresses GCG secretion following both 24 h and 72 h exposure under low and high glucose conditions when compared to controls (Figure S1h,i). These findings indicate that cytokine treatment leads to a global suppression of GCG expression and secretion in α‐cells.

### 3.3 Inflammatory Cytokines Reduce GCG Secretion and Activate Stress Programs in Isolated Islets

**FIGURE 3:**
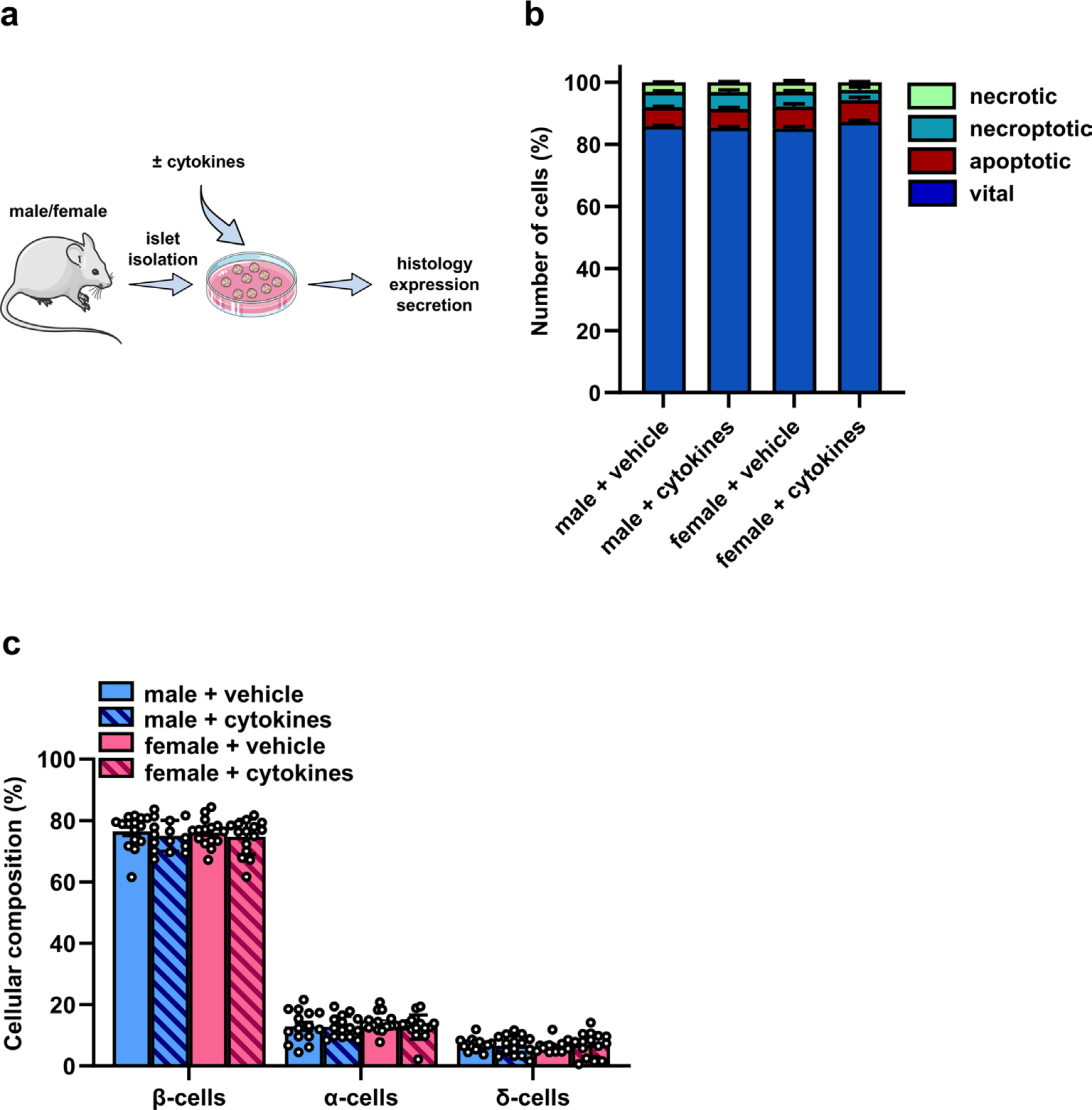
Inflammatory cytokines do not affect the viability of isolated islets. (a) Schematic illustration of the experimental ex vivo setting: Islets were isolated from male and female mice and exposed to a cytokine mix (2 ng/mL IL‐1β, 10 ng/mL TNF‐α and 50 ng/mL IFN‐γ) or the equivalent volume of PBS (vehicle) as control for 24 h. Then, the islets were used for histology, protein and mRNA expression analysis as well as GCG ELISA. (b) Quantitative analysis of propidium iodide/annexin V‐stained cells from isolated male and female islets exposed to a cytokine mix or vehicle for 24 h subdivided in necrotic, necroptotic, apoptotic and vital cells in % of total cell number (*n* = 3 each). (c) Quantitative analysis of insulin‐ (β‐cells), GCG‐ (α‐cells) and SST‐ (δ‐cells) positive cells within isolated male and female islets exposed to a cytokine mix or vehicle for 24 h. Data are expressed in % of all islet cells (*n* = 15 islets each). Mean ± SEM.

**FIGURE 4:**
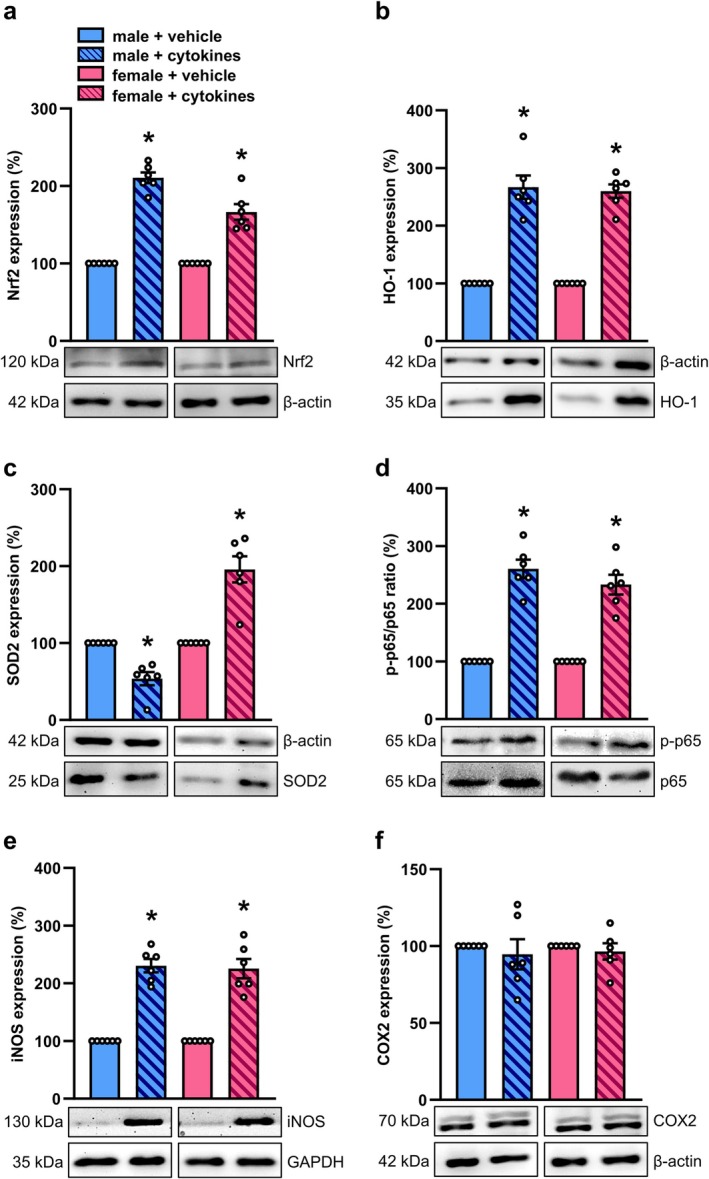
Inflammatory cytokines activate stress programmes in isolated islets. (a–f) Upper panel: Nrf2 (a), HO‐1 (b), SOD2 (c), p‐p65/p65 ratio (d), iNOS (e) and COX2 (f) protein expression in isolated male and female islets exposed to a cytokine mix or vehicle for 24 h. Data are expressed in % of vehicle‐treated male or female islets (*n* = 6 each). Mean ± SEM. *p < 0.05 versus male + vehicle or female + vehicle. Lower panel: Representative Western blots of Nrf2, HO‐1, SOD2, p‐p65/p65, iNOS, COX2, GAPDH and β‐actin expression in whole cell extracts of these islets.

**FIGURE 5:**
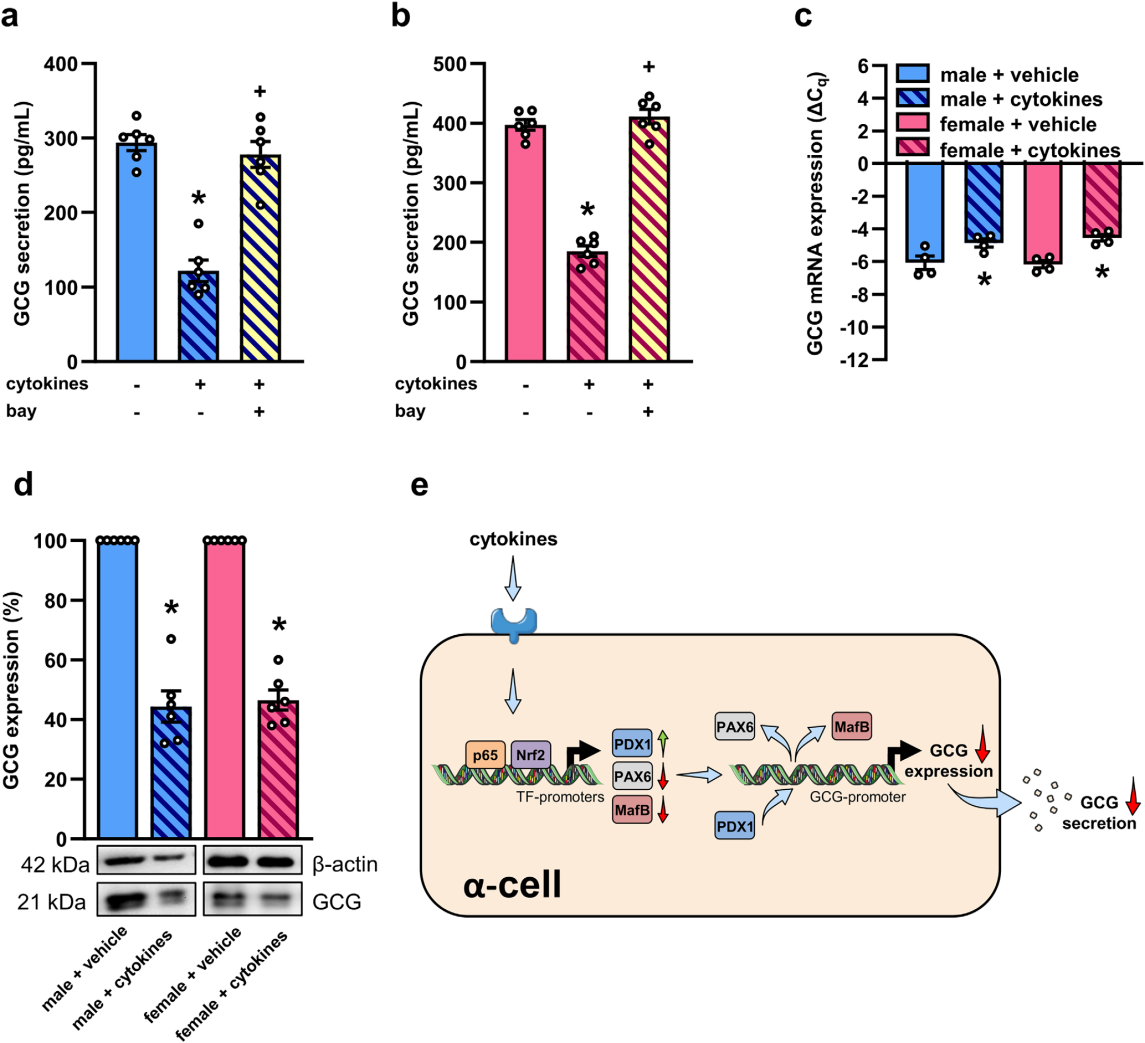
Inflammatory cytokines reduce GCG secretion of isolated islets. (a) GCG secretion (pg/mL) from isolated male islets exposed to vehicle, a cytokine mix or a combination of bay 11‐7082 and cytokine mix (*n* = 6 each). Mean ± SEM. *p < 0.05 versus vehicle. ^+^ *p* < 0.05 versus cytokines. (b) GCG secretion (pg/mL) from isolated female islets exposed to vehicle, a cytokine mix or a combination of bay 11‐7082 and cytokine mix (*n* = 6 each). Mean ± SEM. *p < 0.05 versus vehicle. ^+^ *p* < 0.05 versus cytokines. (c) GCG mRNA expression in isolated male and female islets exposed to a cytokine mix or vehicle for 24 h. Data are expressed in absolute Δ*C*_*q*_ values (*n* = 4 each). Mean ± SEM. *p < 0.05 versus male + vehicle or female + vehicle. (d) Upper panel: GCG protein expression in isolated male and female islets exposed to a cytokine mix or vehicle for 24 h. Data are expressed in % of vehicle‐treated male or female islets (*n* = 6 each). Mean ± SEM. *p < 0.05 versus male + vehicle or female + vehicle. Lower panel: Representative Western blots of GCG and β‐actin expression in whole cell extracts of these islets. (e) Schematic illustration of the underlying molecular mechanism: Cytokines crucially affect GCG secretion by regulating the expression of key transcription factors (TF) PDX1, PAX6 and MafB.

To validate our findings in a more physiological setting, we exposed isolated mouse islets from male and female donors to the identical mix of IL‐1β, TNF‐α and IFN‐γ (Figure 3a). The flow cytometry analyses showed that the cytokines do not induce cell death when compared to controls (Figure 3b). Moreover, the immunohistochemical quantification of endocrine cell composition revealed no differences in α‐, β‐ or δ‐cell fractions between male and female islets (Figure 3c). As observed in αTC1 cells, cytokine exposure also activated stress pathways in the isolated islets (Figure 4a–f). Of interest, female islets exhibited an increased expression of Nrf2, HO‐1 and SOD2, whereas male islets showed elevated Nrf2 and HO‐1 levels but reduced SOD2 expression (Figure 4a–c). Activation of NF‐κB signalling was confirmed by increased p65 phosphorylation and induction of iNOS (Figure 4d,e). COX2 expression did not differ between the groups (Figure 4f). In line with our results of the experiments with αTC1 cells, treatment with the cytokine mix or with the individual cytokines significantly reduced GCG secretion in both male and female islets (Figures 5a,b and S2a,b), which could be abolished by the pre‐treatment of islets with bay (Figure 5a,b). Additional analyses revealed that this reduced GCG secretion is caused by a significant decrease in mRNA and protein expression of GCG (Figure 5c,d). Taken together, in the present study, we demonstrated that cytokines crucially affect GCG secretion by regulating the key transcription factors PDX1, PAX6 and MafB (Figure 5e).

## 4 Discussion

IL‐1β, TNF‐α and IFN‐γ are major proinflammatory cytokines and central mediators of the low‐grade chronic inflammation associated with obesity and T2DM. These cytokines are predominantly secreted by adipose tissue but can also be released by infiltrating immune cells and dysfunctional pancreatic islet cells [16]. In the present study, we used 2 ng/mL IL‐1β, 10 ng/mL TNF‐α and 50 ng/mL IFN‐γ to induce inflammatory stress conditions in vitro, which mimic serum levels of T2DM patients [17, 18]. Previous studies from our group and others demonstrated that exposure to these cytokines promotes islet stress and impairs hormone secretion [5, 7, 19, 20]. Based on these observations, we hypothesised that inflammatory cytokines may also affect pancreatic α‐cell function; however, this aspect has not yet been systematically investigated.

We could show that α‐cells do not undergo cell death when exposed to IL‐1β, TNF‐α and IFN‐γ. However, these cytokines activate the Nrf2 pathway leading to the upregulation of anti‐oxidant defence mechanisms [21, 22, 23]. Accordingly, we observed increased protein levels of HO‐1 and SOD2 in cytokine‐treated α‐cells. These findings suggest that Nrf2 activation may protect α‐cells from cytokine‐induced cytotoxicity. It is well known that cytokine exposure activates the NF‐κB pathway, a key regulator of inflammatory mediators, including iNOS and COX2. Both α‐cells and isolated islets exhibited an increased iNOS expression following cytokine treatment. This observation is consistent with findings by Stancill et al. [24] demonstrating that treatment of islets with IL‐1β and IFN‐γ stimulates iNOS expression in a subset of β‐ and non‐β‐endocrine cells. However, in the present study, we did not observe significant alterations in COX2 levels, although its expression is typically elevated in islets from patients with T2DM as a result of the inflammatory milieu [25]. These contradictory results may be explained by differences in the species origin of the islets, as well as variations in the concentrations and exposure times.

It is well known that adipose tissue of obese patients releases pro‐inflammatory cytokines. This mild and systemic inflammation in combination with chronic elevated levels of blood glucose, amino acids and fatty acids disrupt insulin signalling pathways, acting as a primary driver of insulin resistance [16]. Moreover, studies already reported that under these multifactorial conditions α‐cells no longer respond to insulin [26]. This, in turn, leads to hyperglucagonemia resulting in an increased hepatic glucose production. Based on these findings, it may be conceivable that cytokines increase the expression of GCG. However, we found that islets exposed to IL‐1β, TNF‐α and IFN‐γ exhibit a significantly reduced GCG expression and secretion. This is in line with these results from the group of Leiter [11], who detected a markedly reduced GCG secretion from αTC1 cells exposed to the cytokines IL‐1 and IFN‐γ. We next analysed the transcriptional machinery of GCG gene expression; we detected an upregulated PDX1 mRNA expression in cytokine‐treated cells. Baumel‐Alterzon et al. [27] found reduced levels of PDX1 in pancreatic islets after Nrf2 depletion. Moreover, we have shown that PDX1 represses GCG expression in pancreatic α‐cells by a direct binding to the GCG promoter [28]. Hence, it can be concluded that the herein observed cytokine‐induced Nrf2 signalling is essential for preserving PDX1 expression, which may reduce GCG gene expression in α‐cells. Furthermore, we found a lower PAX6 and MafB expression after cytokine exposure. Studies have already reported that the exposure of islets to IL‐1β, TNF‐α and IFN‐γ decreases the mRNA expression of the transcription factors MafB and PAX6 [4, 29], which has been shown to promote GCG gene expression [30]. Hence, the reduced PAX6 and MafB expression may further contribute to the herein observed decreased GCG gene expression.

Henriksen et al. [31] examined GCG secretion in pancreatic microtissues exposed to cytokines and reported a time‐dependent effect. After short‐term incubation (1 day), GCG expression increased, whereas prolonged exposure (6 days) resulted in decreased expression. In our study, we observed a decrease in GCG release of cells and islets after pre‐treatment with cytokines. These distinct effects after 24 h may be explained by the different cytokine concentrations used. Henriksen et al. [31] exposed pancreatic microtissues to relatively high cytokine concentrations (up to 20 ng/mL IL‐1β, 100 ng/mL TNF‐α and 100 ng/mL IFN‐γ), whereas we used markedly lower cytokine levels (2 ng/mL IL‐1β, 10 ng/mL TNF‐α and 50 ng/mL IFN‐γ) for both α‐cells and isolated islets. Several studies reported that high cytokine levels trigger apoptotic pathways in isolated rodent and human islets [19, 30, 32, 33, 34]. Hence, it is conceivable that high levels of cytokines lead to endocrine dysfunctional cells ultimately resulting in islet cell death.

Sex differences substantially influence the development of T2DM. Men tend to accumulate visceral adipose tissue and the resulting chronic low‐grade inflammation activates pathways, such as NF‐κB, which in turn impairs insulin signalling and promotes insulin resistance [35]. In contrast, women typically exhibit higher amounts of subcutaneous fat and their oestrogens suppress the production of pro‐inflammatory cytokines, such as TNF‐α and IL‐6 [36]. Because inflammatory cytokines diminish the expression and secretion of pancreatic hormones, it is not surprising that women exhibit higher insulin secretion than men [37]. Furthermore, Karlsson et al. [38] reported markedly higher circulating GCG levels in women when compared to men. In line with these observations, we also found a higher GCG secretion in female islets when compared to male islets.

Finally, this study also faces some limitations. First, our findings demonstrating cytokine‐induced alterations in α‐cell function are based on in vitro and ex vivo models. These do not fully recapitulate the complex dysregulated glucose metabolism during the progression of T2DM. Hence, further in vivo studies are required to elucidate how inflammation‐driven alterations in glucagon secretion affect glycemic control. Second, our experiments were conducted exclusively using murine islets as well as murine α‐cells. Given known species‐specific differences in islet biology and cellular responses [39, 40], it will be important to validate our findings in human islets.

Taken together, our novel results not only support the current view that an inflammatory milieu markedly affects the endocrine function of pancreatic islets but also show that inflammatory cytokines activate stress‐response pathways in α‐cells, impairing their transcriptional network controlling glucagon biosynthesis. However, it should be noted that reduced GCG levels are not necessarily beneficial in the context of diabetes, as insufficient GCG secretion may impair the physiological counterregulatory response to hypoglycemia and thereby increase the risk of severe hypoglycemic episodes [41]. In addition, GCG plays an important role inhibiting insulin secretion. Hence, a decreased GCG may further promote hyperglycemia [42, 43].

## Author Contributions

C.B., C.W., M.W.L., E.A., L.P.R. and S.W. designed the research and wrote the manuscript. C.B., S.W., C.W. and E.A. conducted experiments. C.B., S.W., C.W. and E.A. analysed data and interpreted the results. E.A. and S.W. supervised the study. All authors read and approved the final version of the manuscript.

## Funding

Open Access funding enabled and organised by Projekt DEAL.

## Conflicts of Interest

The authors declare no conflicts of interest.

## Supporting information


**Figure S1:** Inhibition of NF‐κB pathway reverses cytokine‐mediated reduced GCG gene and protein expression in α‐cells. (a) Upper panel: iNOS protein expression in αTC1 cells exposed to vehicle, a cytokine mix or a combination of bay 11‐7082 and cytokine mix. Data are expressed in % of vehicle‐treated cells (*n* = 6 each). Mean ± SEM. *p < 0.05 versus vehicle. ^+^ *p* < 0.05 versus cytokines. Lower panel: Representative Western blots of iNOS and β‐actin expression in whole cell extracts. (b–e) PDX1 (b), PAX6 (c), MafB (d) and GCG (e) mRNA expression in αTC1 cells exposed to vehicle, a cytokine mix or a combination of bay 11‐7082 and cytokine mix. Data are expressed in % to vehicle‐treated cells (*n* = 6 each). Mean ± SEM. *p < 0.05 vs. vehicle. ^+^ *p* < 0.05 vs. cytokines. (f) Upper panel: GCG protein expression in αTC1 cells exposed to vehicle, a cytokine mix or a combination of bay 11‐7082 and cytokine mix. Data are expressed in % of vehicle‐treated cells (*n* = 6 each). Mean ± SEM. *p < 0.05 versus vehicle. ^+^ *p* < 0.05 versus cytokines. Lower panel: Representative Western blots of GCG and β‐actin expression in whole cell extracts. (g) GCG secretion (pg/mL) from cells exposed to vehicle, IL‐1β, TNF‐α or IFN‐γ (*n* = 6 each). Mean ± SEM. *p < 0.05 versus vehicle. (h and i) GCG secretion (pg/mL) from cells exposed to vehicle or a cytokine mix for 24 h (h) or 72 h (i) under low (0.5 mM) and high (11 mM) glucose conditions (*n* = 6 each). Mean ± SEM. *p < 0.05 versus vehicle. ^+^ *p* < 0.05 versus cytokines.

**Figure S2:** IL‐1β, TNF‐α or IFN‐γ exert inhibitory effects on GCG secretion of isolated islets. (a) GCG secretion (pg/mL) from isolated male islets exposed to vehicle, IL‐1β, TNF‐α or IFN‐γ (*n* = 6 each). Mean ± SEM. *p < 0.05 versus vehicle. (b) GCG secretion (pg/mL) from isolated female islets exposed to vehicle, IL‐1β, TNF‐α or IFN‐γ (*n* = 6 each). Mean ± SEM. *p < 0.05 versus vehicle.

## Data Availability

All data generated or analysed during this study are included in this published article.
