# Supplementary figures and images for: Inflammatory Cytokines Impair Glucagon Expression and Secretion in Pancreatic α‐Cells
Source: Diabetes Obes Metab. 2026 May 31;28(8):7149–60. doi: 10.1111/dom.70921 (PMC13341363; doi:10.1111/dom.70921)

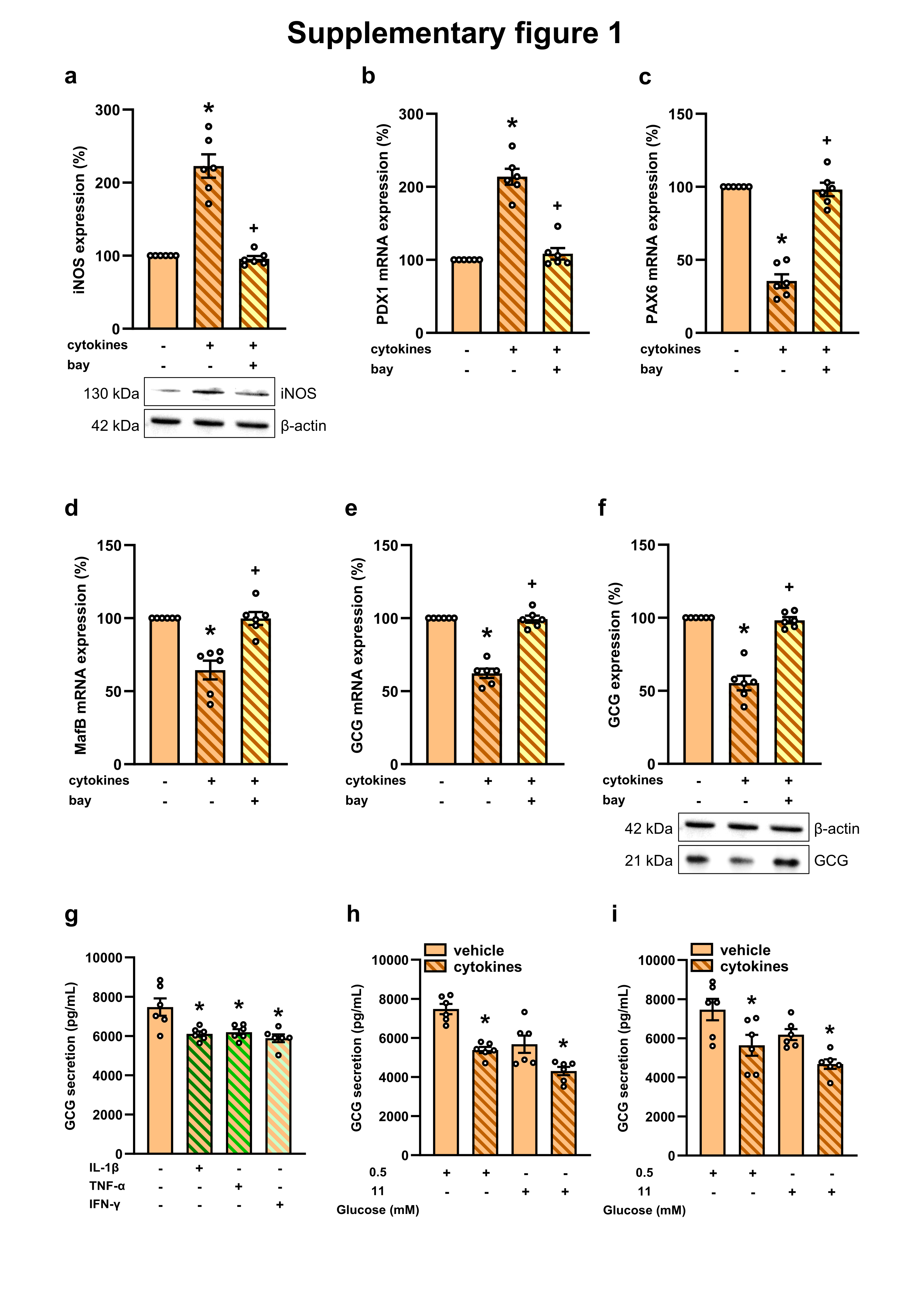

Supplement: Supplementary file 1 — Figure S1: Inhibition of NF‐κB pathway reverses cytokine‐mediated reduced GCG gene and protein expression in α‐cells. (a) Upper panel: iNOS protein expression in αTC1 cells exposed to vehicle, a cytokine mix or a combination of bay 11‐7082 and cytokine mix. Data are expressed in % of vehicle‐treated cells (n = 6 each). Mean ± SEM. *p < 0.05 versus vehicle. + p < 0.05 versus cytokines. Lower panel: Representative Western blots of iNOS and β‐actin expression in whole cell extracts. (b–e) PDX1 (b), PAX6 (c), MafB (d) and GCG (e) mRNA expression in αTC1 cells exposed to vehicle, a cytokine mix or a combination of bay 11‐7082 and cytokine mix. Data are expressed in % to vehicle‐treated cells (n = 6 each). Mean ± SEM. *p < 0.05 vs. vehicle. + p < 0.05 vs. cytokines. (f) Upper panel: GCG protein expression in αTC1 cells exposed to vehicle, a cytokine mix or a combination of bay 11‐7082 and cytokine mix. Data are expressed in % of vehicle‐treated cells (n = 6 each). Mean ± SEM. *p < 0.05 versus vehicle. + p < 0.05 versus cytokines. Lower panel: Representative Western blots of GCG and β‐actin expression in whole cell extracts. (g) GCG secretion (pg/mL) from cells exposed to vehicle, IL‐1β, TNF‐α or IFN‐γ (n = 6 each). Mean ± SEM. *p < 0.05 versus vehicle. (h and i) GCG secretion (pg/mL) from cells exposed to vehicle or a cytokine mix for 24 h (h) or 72 h (i) under low (0.5 mM) and high (11 mM) glucose conditions (n = 6 each). Mean ± SEM. *p < 0.05 versus vehicle. + p < 0.05 versus cytokines. [file DOM-28-7149-s002.tif]

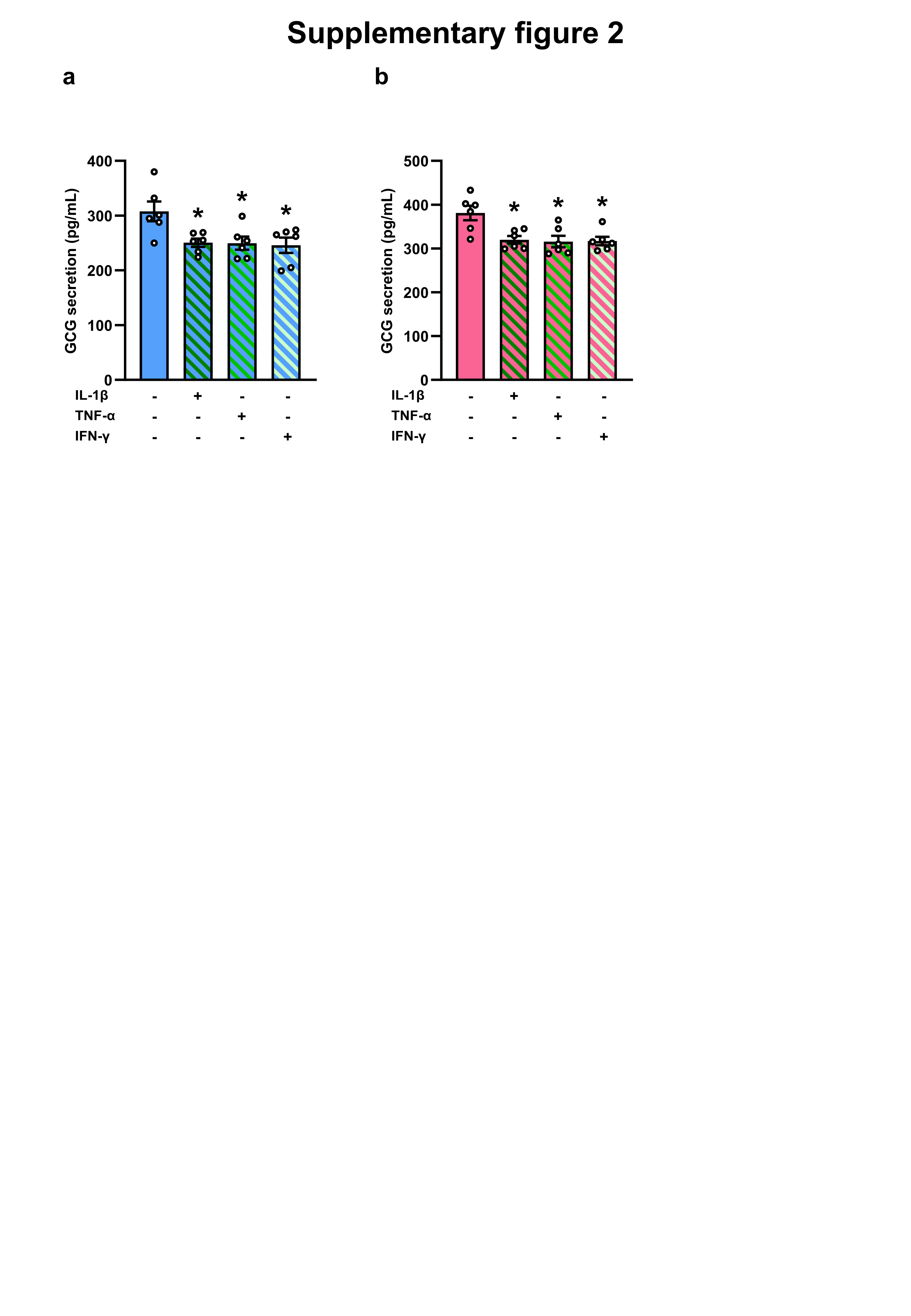

Supplement: Supplementary file 2 — Figure S2: IL‐1β, TNF‐α or IFN‐γ exert inhibitory effects on GCG secretion of isolated islets. (a) GCG secretion (pg/mL) from isolated male islets exposed to vehicle, IL‐1β, TNF‐α or IFN‐γ (n = 6 each). Mean ± SEM. *p < 0.05 versus vehicle. (b) GCG secretion (pg/mL) from isolated female islets exposed to vehicle, IL‐1β, TNF‐α or IFN‐γ (n = 6 each). Mean ± SEM. *p < 0.05 versus vehicle. [file DOM-28-7149-s001.tif]
